# Proteomic Response of Rat Pituitary Under Chronic Mild Stress Reveals Insights Into Vulnerability and Resistance to Anxiety or Depression

**DOI:** 10.3389/fgene.2021.751999

**Published:** 2021-09-17

**Authors:** Fenfang Tian, Dan Liu, Jin Chen, Wei Liao, Weibo Gong, Rongzhong Huang, Liang Xie, Faping Yi, Jian Zhou

**Affiliations:** ^1^ Institute of Neuroscience, Basic Medical College, Chongqing Medical University, Chongqing, China; ^2^ Department of Neurology, The First Affiliated Hospital of Nanchang University, Nanchang, China; ^3^ Statistics Laboratory, ChuangXu Institute of Life Science, Chongqing, China; ^4^ Chongqing Institute of Life Science, Chongqing, China; ^5^ Department of Neurology, The Second Affiliated Hospital of Nanchang University, Nanchang, China

**Keywords:** anxiety, chronic mild stress, depression, proteome, rat pituitary

## Abstract

Chronic stress as one of the most significant risk factor can trigger overactivity of hypothalamic-pituitary-adrenal (HPA) axis in depression as well as anxiety. Yet, the shared and unique neurobiological underpinnings underlying the pituitary abnormality in these two disorders have not been made clear. We previously have established depression-susceptible, anxiety-susceptible and insusceptible groups using a valid chronic mild stress (CMS) model. In this work, the possible protein expression changes in the rat pituitary of these three groups were continuously investigated through the use of the comparative quantitative proteomics and bioinformatics approaches. The pituitary-proteome analysis identified totally 197 differential proteins as a CMS response. These deregulated proteins were involved in diverse biological functions and significant pathways potentially connected with the three different behavioral phenotypes, likely serving as new investigative protein targets. Afterwards, parallel reaction monitoring-based independent analysis found out that expression alterations in *Oxct1*, *Sec24c*, *Ppp1cb*, *Dock1*, and *Coq3*; *Lama1*, *Glb1*, *Gapdh*, *Sccpdh*, and *Renbp*; *Sephs1*, *Nup188*, *Spp1*, *Prodh1*, and *Srm* were specifically linked to depression-susceptible, anxiety-susceptible and insusceptible groups, respectively, suggesting that the same CMS had different impacts on the pituitary protein regulatory system. Collectively, the current proteomics research elucidated an important molecular basis and furnished new valuable insights into neurochemical commonalities and specificities of the pituitary dysfunctional mechanisms in HPA axis underlying vulnerability and resistance to stress-induced anxiety or depression.

## Introduction

Anxiety and depression are two severe and chronic neuropsychiatric illnesses. The prevalences of these disorders are increasing, potentially representing a significant clinical challenge. Mounting evidence suggests that many risk factors are shared between the anxiety and depression disorders such as chronic life stress (Krishnan et al., 2007; Zhou et al., 2016; Jefferson et al., 2020). Chronic stress can result in the adverse health impacts when it increases beyond a certain level, thereby causing anxiety and depression (Chang and Grace, 2014; Tian et al., 2020). However, many individuals can manage the psychological and physical effects of the stressful situations and do not have the disease symptoms (Henningsen et al., 2012). To model the adverse environment factors that affect humans, chronic mild stress (CMS) protocol has been commonly employed to induce anxious-like and depressive-like behaviors in rodent animals (Chang and Grace, 2014; Zhou et al., 2016). To identify the potential biological relationships between CMS and pathological changes, it may be useful to focus on the neurobiological components and processes reflecting adaptive and maladaptive responses to the stress-caused anxiety and depression.

Generally, the clinical symptoms of anxiety and depression are different. However, they are frequently presented simultaneously (Liu et al., 2021; Thorp et al., 2021). Importantly, there are lots of overlaps with respect of the pathophysiology and comorbidity of these two disorders. Considerable data in many clinical and animal researches are usually mixed, thereby confusing our knowledge of the underlying causes and effects of anxiety and depression (Chiba et al., 2012; Lucassen et al., 2016; Oh et al., 2020). In recent years, researchers have attempted to separately investigate non-comorbid individuals to unravel the specificities and commonalities of the two disorders (Lotan et al., 2014; Hamilton et al., 2015; Zhao et al., 2017; Chen et al., 2018). Many studies have demonstrated that the activity of hypothalamic-pituitary-adrenal (HPA) axis is perturbed in these stress-related disorders (Borrow et al., 2016; Delvecchio et al., 2017; Lee and Rhee, 2017). As an integral part of the HPA axis, the pituitary synthesizes and secretes a variety of hormones to mediate a series of biological functions (Yelamanchi et al., 2018). It may be one of the areas most impacted by stress dysregulation in anxiety and depression (Stelzhammer et al., 2015). An increase in the size of the pituitary has also been found in subjects with depression and anxiety through magnetic resonance imaging (Tsigos and Chrousos, 2002; Lorenzetti et al., 2009; Krishnamurthy et al., 2017). To some extent, this reflects an increase in the size and number of corticotropin-releasing hormone (CRH) cells that produce and secrete higher levels of hormones, such as CRH and adrenocorticotrophic hormone (ACTH) (Tsigos and Chrousos, 2002; Krishnamurthy et al., 2017). Despite the morphological and functional abnormalities of the pituitary have been implicated in stress-related anxiety and depression, the corresponding neurobiological molecular basis may remain difference and need to be extensively explored.

Our previous study has demonstrated the three different subpopulations induced by CMS including depression-susceptible (Dep-Sus), anxiety-susceptible (Anx-Sus), and insusceptible (Insus) groups and carried out the comparative proteomic analysis of the rat hippocampal tissues (Tang et al., 2019). In this work, the pituitary tissues from the identical batch of CMS-exposed rats were used to continuously study stress-caused anxiety and depression (Tang et al., 2019). A proteomic approach based on isobaric tags for relative and absolute quantitation (iTRAQ) was utilized to gain unbiased profiling data. Enrichments of Gene Ontology (GO) and Kyoto Encyclopedia of Genes and Genomes (KEGG) analyses were conducted to analyze the main function and the significant pathways of the identified abnormally-expressed proteins. The Search Tool for the Retrieval of Interacting Genes/Proteins (STRING) database and Cytoscape were employed to map protein-protein interaction (PPI) networks. The results help elucidate commonalities and differences of the complex molecular mechanisms that underlie stress resistance and stress-caused anxiety or depression.

## Methods

### Animals and Ethics Statement

Healthy adult male Sprague-Dawley rats (weight, about 250 g; Animal Center of Chongqing Medical University) were used in the present study. All the rats were individually housed in standard laboratory conditions (55 ± 5% relative humidity, 12/12 h light/dark cycle, 21–22°C) with ad libitum feeding. The study protocol was approved by the local Ethics Committee (2017013). All animals were treated according to the National Institutes of Health protocols for the use and care of laboratory animals.

### CMS Rat Model

As previously described (Tang et al., 2019), the 8-weeks CMS protocol was employed to build the rat model. Following exposure to the CMS, the stressed rats were divided into the three groups: 1) Dep-Sus group [assessed by sucrose preference (SP) test and forced swimming (FS) test]; 2) Anx-Sus group [assessed by elevated plus-maze (EPM) test]; and 3) Insus group. Additional non-handled rats acted as the control (Ctrl) group. For a more detailed description, please refer to our previous study (Tang et al., 2019).

### Tissue Isolation and Lysis

After the behavioral assessment, the animals were anesthetized and decapitated and their whole brains were carefully removed on ice. The pituitary tissue was isolated from the rat brain and frozen rapidly in liquid nitrogen and then stored at −80°C in a refrigerator prior to use. For protein extraction, a sample of the pituitary of each animal was added to an SDT buffer composed of 4% SDS, 0.1 M dithiothreitol, 0.1 M Tris–HCl, pH 8.0, and protease inhibitors. The tissues were homogenized and lyzed, the extracted proteins were boiled for 5 min. After centrifugation at 4°C and 40,000 × *g* for 15 min, the supernatants were collected and the protein concentrations were quantified using Pierce bicinchoninic acid assay kit.

### Digestion of Pituitary Proteins and iTRAQ Labeling

Following our previously described procedure (Gong et al., 2021), the protein samples were in parallel digested using filter-aided sample preparation (FASP). In this method, an ultrafiltration filter (10 kD cutoff) was used for effective digestion. In brief, UA buffer (8 M urea, 0.15 M Tris-HCl, pH 8.0) was added to each sample. The sample was transferred to an ultrafiltration centrifuge tube and then centrifuged, and washed again with UA buffer. Subsequently, 0.05 M iodoacetamide in UA buffer was added to the filter. The protein mixture was incubated and alkylated for 30 min at room temperature in the dark. The filter unit was centrifuged and then washed twice with UA buffer. Finally, trypsin solution was added and digested at 37°C overnight. The resulting peptides were collected as a filtrate and then dried in a Speed Vac.

### High-pH Reversed-Phase Liquid Chromatography (RPLC) Fractionation and Liquid Chromatography-Tandem Mass Spectrometry (LC-MS/MS)

**FIGURE 1:**
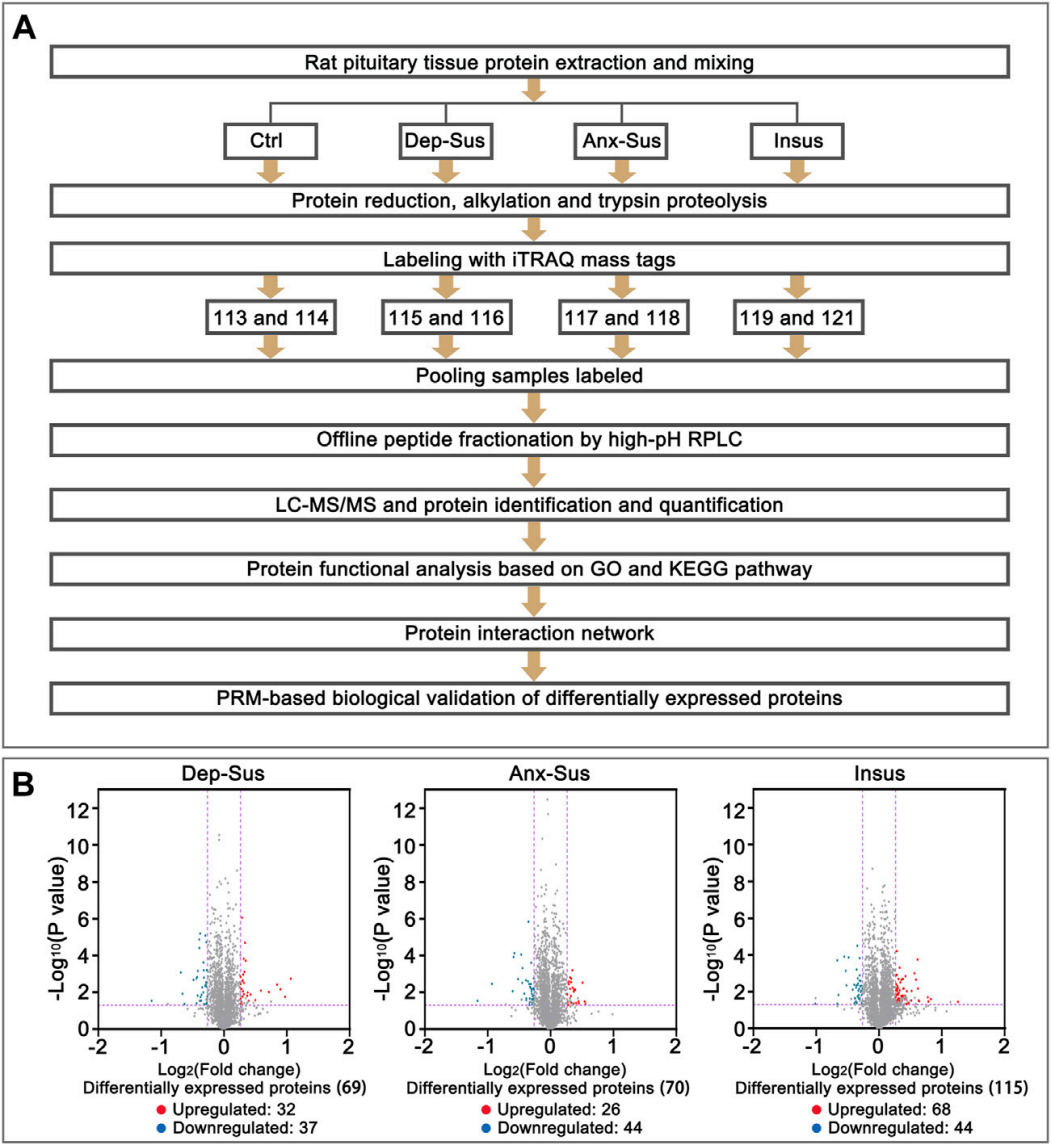
Comparative analysis of the pituitary proteomic response of the rats under chronic mild stress (CMS). **(A)** Schematic representation of quantitative proteomics analysis of the control (Ctrl), depression-susceptible (Dep-Sus), anxiety-susceptible (Anx-Sus) and insusceptible (Insus) groups. **(B)** Volcano plot of the protein expression changes in the three groups. In volcano plot, the red plot represented up-regulated proteins, and the blue plot represented down-regulated proteins. The x-axis shows the log2-transformed average fold change. The y-axis shows the negative log10-transformed *p*-value.

The tryptic peptides were labeled with eight-plex iTRAQ reagents according to the protocol of the manufacturer. The reagents 113–121 were used to label the eight samples from the three stressed and the Ctrl groups, as depicted in Figure 1A. Each used sample was obtained from 2 to 3 rats in each group (Lenselink et al., 2015). Subsequently, the eight labeled samples were pooled and preliminarily separated using high-pH RPLC. Briefly, the peptides were dissolved with buffer A (5% acetonitrile, 0.01 M ammonium formate, pH 10.0) and fractionated through linear elution in a gradient of 5–38% buffer B (90% acetonitrile (ACN), 0.01 M ammonium formate, pH 10.0) for 80 min at 300 μL/min. A total of sixteen fractions were collected, desalted and dried for the subsequent LC-MS/MS analysis.

The peptides in each fraction were re-dissolved in 0.1% formic acid, and delivered into Thermo Scientific Easy-nLC 1200 system coupled with a nanoViper C18 trap column (3 μm, 100 Å). The peptide mixtures were trapped and then desalted using 100% solvent A (0.1% formic acid). Afterward, the peptides were eluted with 8–38% solvent B (80% ACN/0.1% formic acid) for 50 min, and separated with an analytical column (50 μm × 150 mm, 3 μm-C18 100 Å). Q-Exactive Orbitrap mass spectrometer equipped with a Nano Flex ion source (ThermoFisher) was used for the MS analysis (interface heater temperature, 275°C; ion spray voltage, 1.9 kV). The tandem MS data were acquired through the use of a data-dependent acquisition mode along with full MS scans. The acquisition range was 350–1,200 m/z for the MS1 and 110–1,200 m/z for the MS2. For the information acquisition, survey scans were acquired in 250 ms and up to 14 product ion scans (50 ms) were collected. Those MS spectra along with charge state 2–4 were selected and subjected to fragmentation using higher-energy collision dissociation, and dynamic exclusion for selected precursor ions was set to 25 s.

### Protein Identification and Quantification

Raw files were processed and searched using the Sequest HT search engine embedded into Proteome Discoverer software 2.1 (ThermoFisher) against the UniProt Rat database. The following search parameters were set: monoisotopic mass values, fragment mass tolerance at 0.05 Da and precursor mass tolerance ± 10 ppm, trypsin as the enzyme, and allowing up to 2 missed cleavages. Fixed modifications were defined as iTRAQ labeling and carbamidomethylation of Cys; Oxidation on Met, acetylation on protein N-term, deamidation on Asn and Gln, and Pyro-Glu were specified as a variable modification. The decoy database pattern was set as the reversed version of the target database. All reported data were based on 99% confidence for peptide identification as determined by a false discovery rate (FDR) of lower than 1%. Relative ratios of identified peptides among labeled samples were computed using relative peak intensities of released iTRAQ reporter ions in each of the MS/MS spectra, and introduced into Excel spreadsheet for manual treatment. Then, the ratios of all identified proteins were analyzed *via* a two-tailed Student’s t-test. Those proteins with 1.2-fold expression alterations and *p*-values lower than 0.05 could be considered as significantly different. The raw data have been deposited to the ProteomeXchange Consortium (http://proteomecentral.proteomexchange.org) *via* the iProX partner repository with the dataset identifier PXD025429 (Ma et al., 2019).

### Bioinformatics

GO analyses including biological processes (GO-BP), molecular functions (GO-MF), and cellular components (GO-CC) were conducted through the use of the OmicsBean tool (http://www.omicsbean.cn/). KEGG (http://www.genome.jp/kegg/) was used to identify the significant pathways with *p*-values of lower than 0.1 following the previously described procedure (Yu et al., 2017). Moreover, the STRING database and Cytoscape were used to construct PPI networks following the previously reported protocol (Gong et al., 2021).

### Parallel Reaction Monitoring (PRM) MS Assay

Following the iTRAQ-based proteomics experiment, extraction and digestion of the pituitary proteins were performed. The resulting peptides were analyzed using the Q-Exactive Orbitrap mass spectrometer. A normalized collision energy of 28 was used for the fragmentation of the peptides, and the resulting fragments were analyzed at a resolution of 35,000. The acquired raw data were analyzed *via* the Proteome Discoverer tool. The MS data were further processed using the analysis software Skyline 19.1 (ThermoFisher). The statistical analysis of the data were performed using Student’s t tests of SPSS software. The data were presented as means ± standard error (SE). The difference was considered to be statistically significant when *p*-values lower than 0.05.

## Results

### iTRAQ-Based Proteomics Analysis of the Rat Pituitary Under the CMS

In the present work, our used pituitary tissue samples were from the identical batch of the stressed animals in our recently published paper (Tang et al., 2019). Briefly, the stress-induced depressive-like behavior including anhedonia and behavioral despair were firstly assessed through the use of the SP and FS tests. Meanwhile, we also utilized the EPM test for indexing the anxious-like symptom. Based on these testing data, a subset of the Dep-Sus, Anx-Sus, Insus, and Ctrl groups was finally obtained. Overall, these results indicated that we could effectively utilized the CMS model to investigate the neurobiological processes associated with the resistance and vulnerability of stress-related anxious or depressive disorders.

Next, we investigated the effects of CMS on the expression of the rat pituitary proteins through the use of iTRAQ-based quantitative proteomics analyses (Figure 1A). In this experiment five animals per group were used, and the pituitary proteins from 2 to 3 rats were equally pooled for each sample (Lenselink et al., 2015). Matching to the UniProt database, within the Ctrl, Dep-Sus, Anx-Sus and Insus groups, totally 3,601 non-redundant proteins were identified and quantified based on the FDR lower than 0.01. The iTRAQ-based protein expressions that changed greater than 1.2-fold and *p*-values lower than 0.05 *versus* the values for the Ctrl group were deemed to be significantly different. Overall, 197 proteins were found to exhibit a significant differential expression in the three groups (Supplementary Table S1). Here, the proteome profile of the pituitary was contrasted with that of the hypothalamus from our previous work (Gong et al., 2021) (Supplementary Figure S1). Despite the profile of hypothalamus and pituitary was similar based on a 60–70% overlap of the total quantified proteins, the differential protein sets in each area exhibited considerably divergent. This suggested that there were different proteome responses to stress in these two areas.

### Functional and Network Characterization of CMS-Responsive Differential Proteins

**FIGURE 2:**
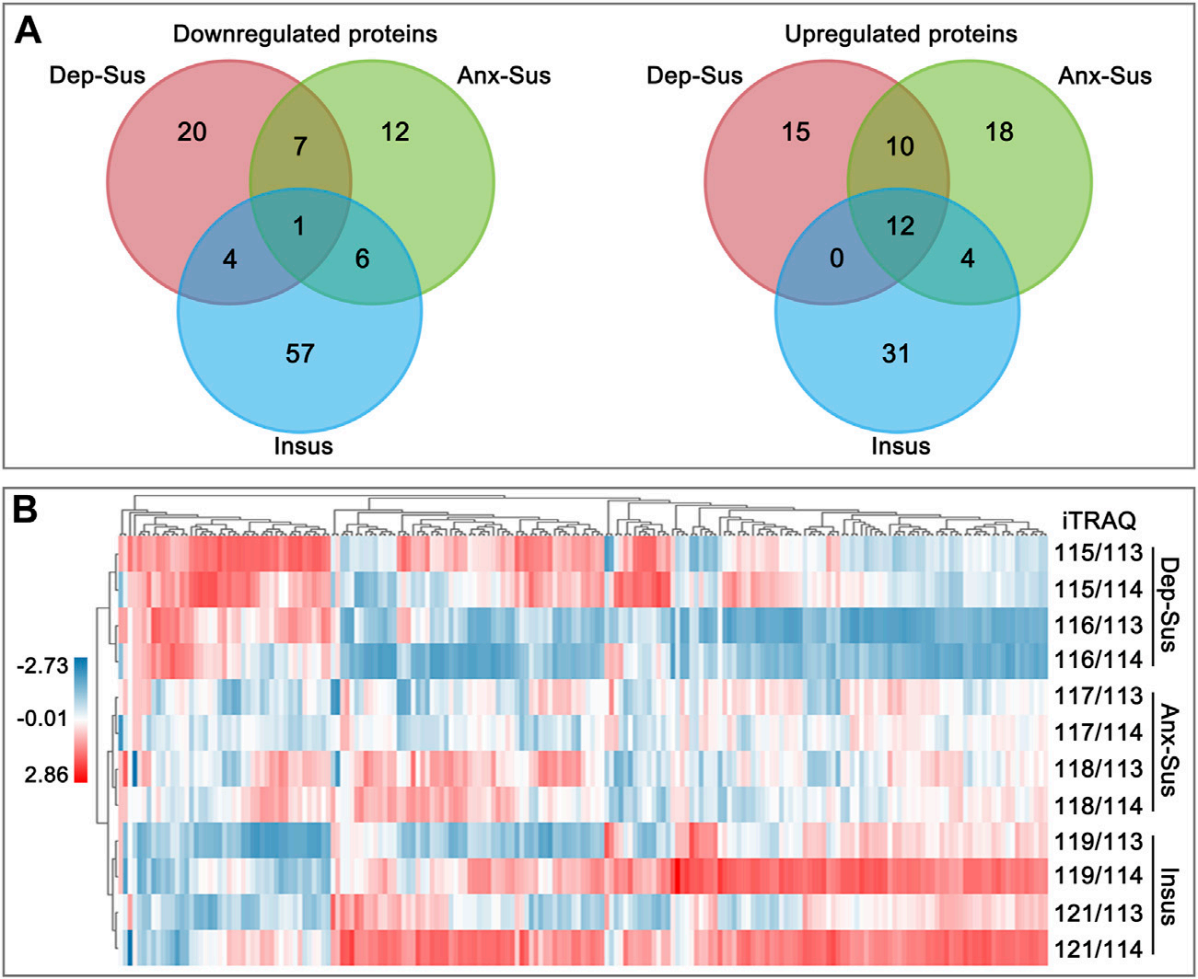
Analysis of the deregulated pituitary proteins identified in the depression-susceptible (Dep-Sus), anxiety-susceptible (Anx-Sus), and insusceptible (Insus) groups. **(A)** Venn diagrams of the deregulated proteins in the three stressed groups. **(B)** Heatmap of the deregulated proteins in the three groups. Higher expressions were indicated by red and lower by blue. The expression levels were shown with various color intensities. In the color bar the log2 scale was used.

The pituitary site-specific proteome signature of the CMS-exposed rats unraveled 37 downregulated and 32 upregulated proteins in the Dep-Sus group, 44 downregulated and 26 upregulated proteins in the Anx-Sus group, and 44 downregulated and 68 upregulated in the Insus group (Figure 1B). In the two stress-susceptible cohorts, 30 proteins were seen to be similarly deregulated, potentially representing the commonality of stress-induced anxiety and depression. Among the susceptible and the insusceptible groups, 27 similarly deregulated proteins were seen and might sever as a consequence of stress exposure (Figure 2A). To sum up, as many as 78% of these deregulated proteins were uniquely connected with the three phenotypes, which demonstrated that the three stressed cohorts had specific protein expression disturbances as a response of stress. Further, based on the unsupervised hierarchical clustering analysis, the expression profile of the 197 deregulated proteins were divided into three different units, to some extent suggesting the three specific CMS responses (Figure 2B).

**FIGURE 3:**
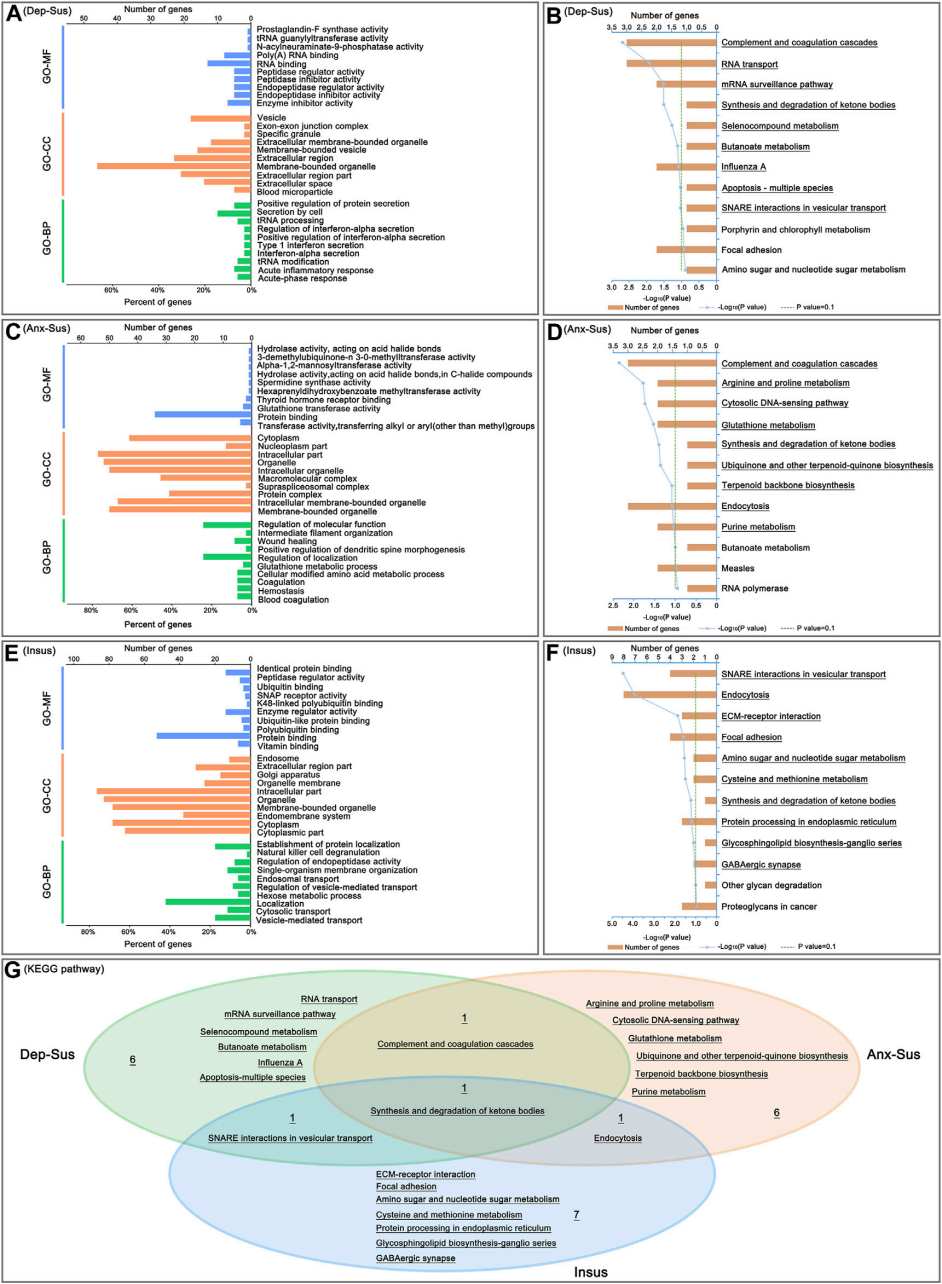
Analysis of Gene Ontology (GO) and Kyoto Encyclopedia of Genes and Genomes (KEGG) pathway enrichments. The top ten enriched GO biological process (GO-BP), cellular component (GO-CC) and molecular function (GO-MF) terms of the deregulated pituitary proteins from the depression-susceptible (Dep-Sus, **A**), anxiety-susceptible (Anx-Sus, **C**) and insusceptible (Insus, **E**) groups were indicated. Meanwhile, the significantly overrepresented KEGG pathway terms from the Dep-Sus **(B)**, Anx-Sus **(D)**, and Insus **(F)** groups were shown with underscores. The x-axis represented the negative log10-transformed *p*-value. **(G)** Venn diagram displaying unique and common significantly-enriched pathways among the three groups.

We carried out GO classification and pathway enrichment of the deregulated proteins through the use of the OmicsBean software, for a better understanding of the significant protein functions and biological pathways correlated with the three behavioral phenotypes. The 69 deregulated proteins in the Dep-Sus group were subjected to enrichment analyses of the GO and KEGG pathways. Total 377, 85, 82, and 9 terms in the GO-BP, GO-CC, GO-MF, and KEGG pathways were significantly overrepresented (Supplementary Table S2). The ten top enriched GO terms are shown in Figure 3A. According to the GO-BP annotations, many deregulated proteins were associated with acute-phase and inflammatory responses, tRNA modification and processing, interferon-alpha, type 1 interferon and protein secretion and regulation. The GO-CC annotations showed that these differential proteins were mainly located in blood microparticle, extracellular region and organelle, and membrane-bounded organelle and vesicle. According to the GO-MF annotations, most of proteins were involved in enzyme inhibitor, peptidase inhibitor and regulator activity, and RNA binding. In pathway enrichment analyses, the deregulated proteins were mainly related to complement and coagulation cascades, RNA transport, mRNA surveillance pathway, synthesis and degradation of ketone bodies, metabolism, apoptosis and SNARE interactions in vesicular transport (Figure 3B).

At the same time, enrichments of GO annotations and KEGG pathways of the 70 deregulated proteins in the Anx-Sus group were carried out. There were 388 GO-BP, 84 GO-CC, 82 GO-MF, and 9 KEGG pathway terms overrepresented. The top 10 enriched GO terms are indicated in Figure 3C. The GO-BP annotations displayed that the majority of proteins were associated with coagulation, hemostasis, and amino acid and glutathione metabolic processes. According to the GO-CC annotations, the differential proteins were mainly found in membrane-bounded and intracellular organelle, protein, supraspliceosomal and macromolecular complex, and nucleoplasm and cytoplasm parts. The GO-MF annotations indicated that most proteins were involved in enzyme activity, protein and thyroid hormone receptor binding. According to pathway enrichment analyses, the deregulated proteins were primarily implicated in complement and coagulation cascades, metabolism and biosynthesis, cytosolic DNA-sensing pathway, and endocytosis (Figure 3D).

Afterwards, GO annotation and KEGG pathway enrichments of the 115 deregulated proteins in the Insus group were also conducted. There were 516 GO-BP, 116 GO-CC, 104 GO-MF, and 10 KEGG pathway terms overrepresented. The top ten enriched GO terms are shown in Figure 3E. According to the GO-BP annotations, most of the differential proteins were involved in vesicle-mediated, cytosolic and endosomal transport and regulation, localization and metabolic process, and GO-CC category analysis showed that the majority of the deregulated proteins located in the cytoplasmic and intracellular parts, endomembrane system, organelle and endosome. The GO-MF annotations predicted that most of the proteins were engaged in vitamin, protein and ubiquitin binding, and enzyme and peptidase regulator, SNAP receptor activities. The pathway enrichment analyses uncovered that the deregulated proteins were mainly enriched in SNARE interactions in vesicular transport, endocytosis, ECM-receptor interaction, focal adhesion, metabolism and biosynthesis, and GABAergic synapse (Figure 3F).

Interestingly, of these significantly-enriched KEGG pathways, there were one shared terms among the three cohorts (Figure 3G). Meanwhile, we could see the two common pathways between the two susceptible cohorts. Importantly, the 6, 6 and 7 pathways were seen to be uniquely related to the Dep-Sus, Anx-Sus and Insus groups, respectively, potentially suggesting the three different neurobiological response to the identical CMS.

**FIGURE 4:**
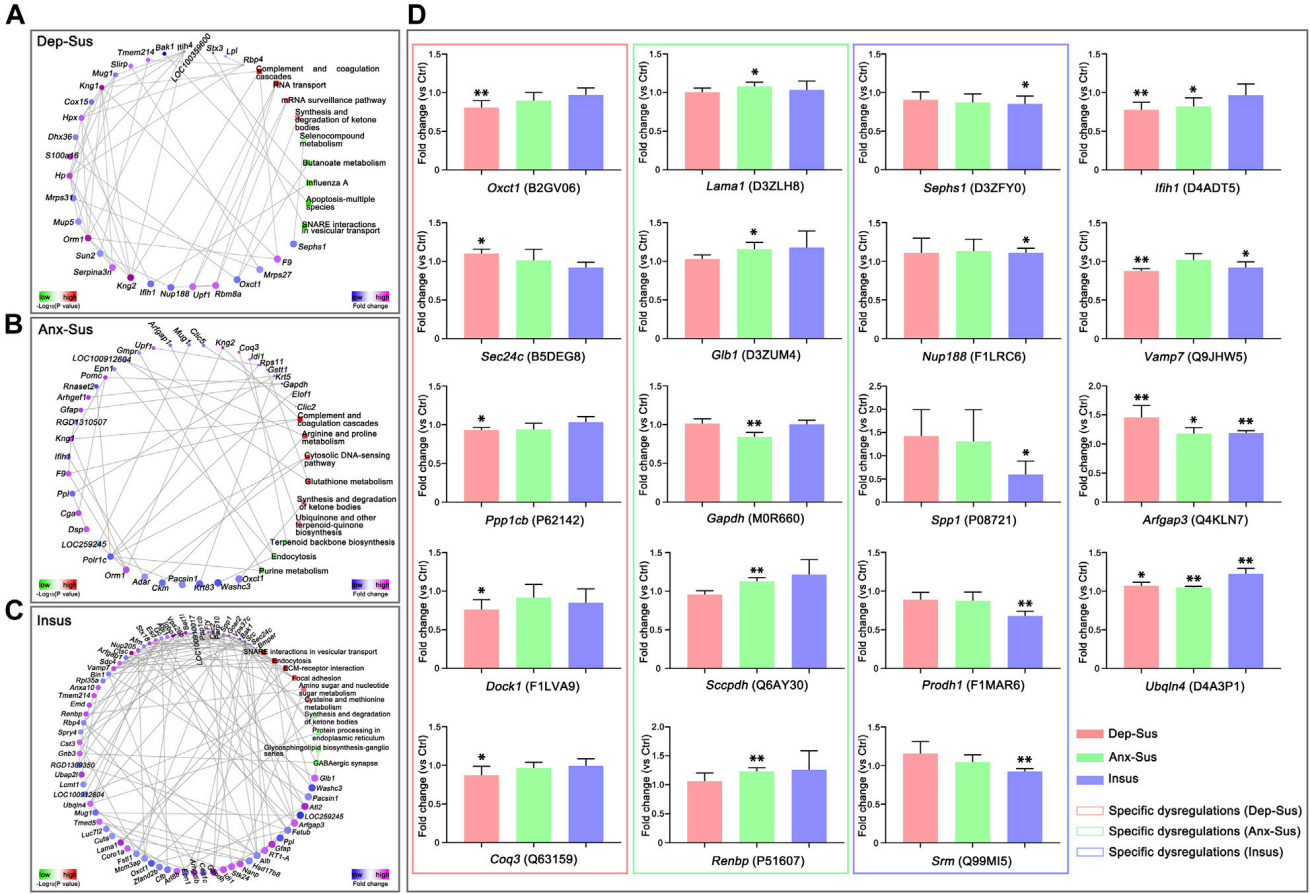
Protein–protein interaction (PPI) network and parallel reaction monitoring (PRM) analyses of the deregulated pituitary proteins of the three groups. The PPI networks of depression-susceptible (Dep-Sus, **A**), anxiety-susceptible (Anx-Sus, **B**), and insusceptible (Insus, **C**) were built based on fold changes of protein expression, PPIs and Kyoto Encyclopedia of Genes and Genomes (KEGG) pathway enrichments. Circular nodes represented proteins/genes and rectangles represented KEGG pathway terms. Lower *p*-value was indicated in blue and higher *p*-value in yellow. **(D)** PRM analysis of the deregulated proteins in the three stressed groups when compared to the control (Ctrl). *Oxct1*, *Sec24c*, *Ppp1cb*, *Dock1*, *Coq3*, *Lama1*, *Glb1*, *Gapdh*, *Sccpdh*, *Renbp*, *Sephs1*, *Nup188*, *Spp1*, *Prodh1*, *Srm*, *Ifih1*, *Vamp7*, *Arfgap3*, and *Ubqln4* were determined on the pituitary protein extracts of the rats. The relative abundance of target proteins among sample groups were compared based on the abundance of the corresponding peptides. *n* = 5 per group, *p < 0.05, **p < 0.01.

Furthermore, we also focused on the proteome-inferred PPI networks in the Dep-Sus, Anx-Sus and Insus groups, as shown in Figures 4A–C. The PPI network maps of the three stressed groups were built through the use of the deregulated proteins correlated with the significant pathways. 29, 36, and 71 deregulated proteins were identified to be several important factors based on the unified conceptual framework of the three networks from the Dep-Sus, Anx-Sus and Insus groups, respectively. As expected, these networks unraveled close relationships between the deregulated proteins and the significantly enriched pathways, thereby furnishing a useful and valuable interactome unit connected with the three different behavioral phenotypes.

### PRM Analysis of CMS-Response Proteins

In this work, the PRM technique was used to further independently validate nineteen abnormally-expressed proteins of interest involved in the significant biological functions and pathways. On the whole, the PRM data mirrored the iTRAQ results (Supplementary Figure S2). As illustrated in other proteomics work (Abdi et al., 2006; Cheng et al., 2011; Xu et al., 2012; Wu et al., 2019), some discrepancies existed between the iTRAQ and PRM data. Except as the assay difference of these two approaches, another probable reason was the additional mixing step in the iTRAQ experiment (Xu et al., 2012; Wu et al., 2019). Compared with the Ctrl group, the expressions of *Oxct1*, *Ppp1cb*, *Dock1*, and *Coq3* were significantly down-regulated while *Sec24c* was up-regulated in the Dep-Sus group; the expressions of *Gapdh* was significantly down-regulated whereas *Lama1*, *Glb1*, *Sccpdh*, and *Renbp* were up-regulated in the Anx-Sus group; the expressions of *Sephs1*, *Spp1,* and *Srm* were significantly down-regulated whereas *Nup188* and *Prodh1* were up-regulated in the Insus group (Figure 4D). In addition, the expression level of *Ifih1* was displayed to be significantly reduced in both the Dep-Sus and Anx-Sus groups as compared to the Ctrl group. The reduced level of *Vamp7* in both the Dep-Sus and Insus groups, and the elevated level of *Arfgap3* and *Ubqln4* in the three stressed groups were observed as contrasted with the Ctrl group.

## Discussion

Chronic stress is the most major factor among many factors that may cause psychiatric illnesses, including anxiety and depression (Henningsen et al., 2012; Chang and Grace, 2014). This is largely due to the means through which the stress affects the function of the HPA axis (Tsigos and Chrousos, 2002). A valid CMS paradigm was thus commonly employed to cause anxious-like and depressive-like behaviors of rats (Henningsen et al., 2012; Chang and Grace, 2014). Previously we constructed the CMS model to gain the three different phenotypes (Dep-Sus, Anx-Sus, and Insus) of the rats through assessment the behavior performance (Tang et al., 2019). This stress model provided a useful means for assay of common and specific neurochemical characteristics of resistance and susceptibility to anxiety or depression. Profiling the phenotype-related protein expressions may lead to new molecular insights into translational research of depression and anxiety.

To discover the phenotype-related protein deregulations, we compared the expression of proteins in the pituitary of the rats exposed to CMS using iTRAQ-based proteomics analyses. There is a total number of 197 deregulated proteins found in the pituitary of Dep-Sus, Anx-Sus, and Insus rats. The overlapped protein deregulations between the Dep-Sus and Anx-Sus groups likely reflected the shared protein expression patterns of anxiety and depression. Those similar deregulations between the Insus and Dep/Anx-Sus groups could be considered to be a general response to CMS. Interestingly, the specifically deregulated protein expressions in the three stressed cohorts suggested potential differences among the stress-induced behavioral phenotypes. The specific protein dysfunctional profiles were further exhibited and evidenced through the use of the clustering analysis.

Subsequently, some potentially affected biological processes and pathways in the pituitary tissue uniquely associated to stress-induced depressive-like and anxious-like behaviors and stress resistance were found through integrated analysis of the proteomics and bioinformatics. The analysis of biological pathways indicated that the deregulated proteins were significantly enriched for complement and coagulation, ketone bodies, vesicular transport, and metabolism dysfunctions in the Dep-Sus group, complement and coagulation, metabolism and endocytosis deregulations in the Anx-Sus group, and vesicular transport, endocytosis, metabolism and synapse repercussions in the Insus group. Importantly, many significant pathways were found to be distinctly connected with the three phenotypes, which reflected differences in active biological processes and events that happened in these stressed cohorts. The further network mapping unraveled the protein deregulation systems and likely offered some useful clues correlated with resistance and susceptibility to stress-caused anxiety or depression.

In this work, we further utilized PRM-based quantitative method to independently validate the nineteen abnormally-expressed proteins involved with the remarkable biological functions and pathways. The results indicated that *Oxct1*, *Sec24c*, *Ppp1cb*, *Dock1*, and *Coq3* were distinctly deregulated in the pituitary of the Dep-Sus group, whereas *Lama1*, *Glb1*, *Gapdh*, *Sccpdh*, and *Renbp* were distinctly deregulated in the Anx-Sus group. These specific alterations suggested that the same stimuli could lead to the different molecular response and neurobiological processes in the pituitary, thereby triggering the depression and anxiety behaviors. Meanwhile, we observed that *Sephs1*, *Nup188*, *Spp1*, *Prodh1*, and *Srm* were distinctly deregulated in the Insus group, suggesting a potential positive way to dealing with the stress-caused pituitary protein deregulations for stress protection and behavioral adaptation (Krishnan et al., 2007; Zhou et al., 2016).

We found that these PRM-determined phenotype-specific deregulated proteins were mainly involved in the metabolism, focal adhesion, protein processing and RNA transport. *Oxct1*, *Coq3*, *Glb1*, *Sccpdh*, *Gapdh*, *Renbp*, *Sephs1*, *Prodh1*, and *Srm* are involved in a wide range of principal metabolic pathways. In the Dep-Sus group, specific dysregulations of *Oxct1* and *Coq3* would result in the abnormalities of synthesis and degradation of ketone bodies, and ubiquinone and other terpenoid-quinone biosynthesis. In the Anx-Sus group, the aberrations of *Glb1* and *Sccpdh* were important for glycosphingolipid and glycolipid biosynthetic processes, and potentially affected the formation of lipid (Dakik et al., 2021). In the Insus group, *Sephs1*, *Prodh1,* and *Srm* have also been reported to participate in some critical metabolic pathways, such as amino sugar and nucleotide sugar metabolism, selenocompound metabolism, and arginine and proline metabolism. More importantly, these metabolisms were generally considered as a significant source of energy supply. The regulatory abnormality of multiple metabolic processes in the pituitary would lead to a negative or positive energy balance of the HPA axis (Nieuwenhuizen and Rutters, 2008; Harris, 2015). Furthermore, *Ppp1cb*, *Dock1*, *Lama1*, and *Spp1* were found to be involved in focal adhesion pathway. These neural cell adhesion molecule might be vital for the neuronal plasticity of stress-induced disorders (Cotman et al., 1998; Ditlevsen et al., 2008). Moreover, dysregulation of *Sec24c* in the Dep-Sus group might affect cell surface levels of the serotonin transporters and thus be linked to depression (Sucic et al., 2011). Meanwhile, we also noted the underexpression of *Ifih1* in both the two susceptible groups, which probably was an important pathological clue for depression and anxiety. In our present work, changes in the expressions of proteins involved in multiple significant biological functions and pathways especially metabolism were identified in the pituitary of the stressed rats, it would be interesting to further explore the possible complex mechanisms behind these stress-induced deregulations pointing to the HPA axis dysfunction in depression and anxiety.

## Conclusion

In this study, we determined the impacts of CMS on the rat pituitary proteome *via* iTRAQ-based and PRM-based quantitative approaches. We found out some candidate pituitary proteins that were likely linked to resistance and susceptibility to CMS-induced depression or anxiety and thus furnished new valuable insights into the stress-affected molecular deregulations in the chronically stressed groups. The current proteomic research can serve as an important molecular underpinning, and help to better understand similarities and differences of the pituitary dysfunction mechanisms in the HPA axis that underlie stress resistance and stress-caused anxiety or depression.

## Data Availability

The datasets presented in this study can be found in online repositories. The names of the repository/repositories and accession number(s) can be found in the article/Supplementary Material.
