# Supplementary material for: Proteomic Response of Rat Pituitary Under Chronic Mild Stress Reveals Insights Into Vulnerability and Resistance to Anxiety or Depression
Source: Front Genet. 2021 Sep 17;12:751999. doi: 10.3389/fgene.2021.751999 (PMC8484759; doi:10.3389/fgene.2021.751999)
Supplement: Supplementary file 3 [file DataSheet1.docx]

**Supplementary material**

**Proteomic response of rat pituitary under chronic mild stress reveals insights into vulnerability and resistance to anxiety or depression**

***Fenfang Tian^1†^, Dan Liu^1†^, Jin Chen^1,2†^, Wei Liao^1^, Weibo Gong^1^, Rongzhong Huang^3,4^, Liang Xie^1,5*^, Faping Yi^1*^, Jian Zhou^1*^***

*^1^Institute of Neuroscience, Basic Medical College, Chongqing Medical University, Chongqing 400016, China*

*^2^Department of Neurology, The First Afﬁliated Hospital of Nanchang University, Nanchang 330006, China*

*^3^Statistics Laboratory, ChuangXu Institute of Life Science, Chongqing 400016, China*

*^4^Chongqing Institute of Life Science, Chongqing 400016, China*

*^5^Department of Neurology, The Second Afﬁliated Hospital of Nanchang University, Nanchang 330006, China*

^†^These authors contributed equally to this work.

*To whom correspondence should be addressed:

Faping Yi and Jian Zhou

Institute of Neuroscience, Basic Medical College, Chongqing Medical University, 1 Yixueyuan Road, Yuzhong District, Chongqing 400016, China. Tel: +86-23-68485763. E-mail addresses: 100506@cqmu.edu.cn (F. Yi), zhoujian@cqmu.edu.cn (J. Zhou).

Liang Xie

Department of Neurology, The Second Affiliated Hospital of Nanchang University, 1 Minde Road, Nanchang 330006, Jiangxi, China. E-mail address: xl580122@163.com (L. Xie).

**Supplementary material**

**Supplementary Figure S1.** Comparisons of the proteome profiles of the hypothalamus and pituitary. (A) Venn diagram displaying the number of the total proteins quantified in each brain area. (B, C and D) The diagrams showing the number of differential proteins in the depression-susceptible (Dep-Sus, B), anxiety-susceptible (Anx-Sus, C) and insusceptible (Insus, D). Hyp: hypothalamus; Pit: pituitary.

**Supplementary Figure S2.** Comparison between isobaric tags for relative and absolute quantitation (iTRAQ)-based and parallel reaction monitoring (PRM)-based data from the depression-susceptible (Dep-Sus), anxiety-susceptible (Anx-Sus), insusceptible (Insus) groups relative to the control (Ctrl).

**Supplementary Table S1.** Protein identifications from the depression-susceptible (Dep-Sus), anxiety-susceptible (Anx-Sus) and insusceptible (Insus) groups through the use of the isobaric tag for relative and absolute quantitation (iTRAQ)-based proteomic approach. The deregulated proteins were shown in green.

**Supplementary Table S2.** Complete enriched terms in gene ontology (GO) biological process (GO-BP), cellular component (GO-CC), molecular function (GO-MF), and Kyoto Encyclopedia of Genes and Genomes (KEGG) pathway of the deregulated proteins from the depression-susceptible (Dep-Sus), anxiety-susceptible (Anx-Sus), and insusceptible (Insus) groups. The significantly enriched terms were shown in green.


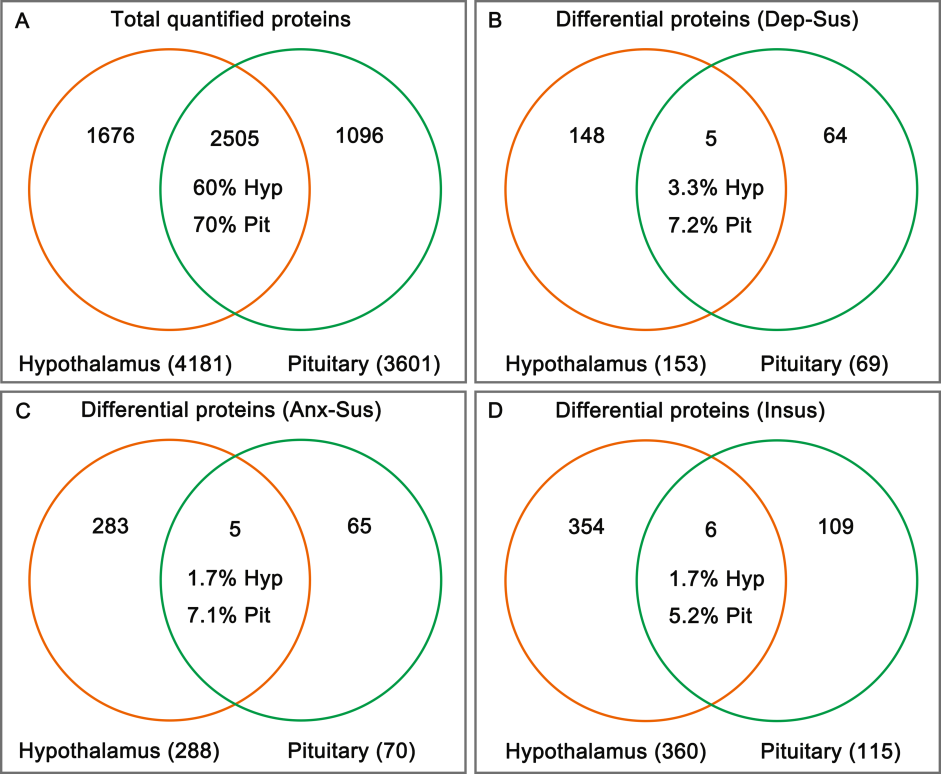


**Supplementary Figure S1**


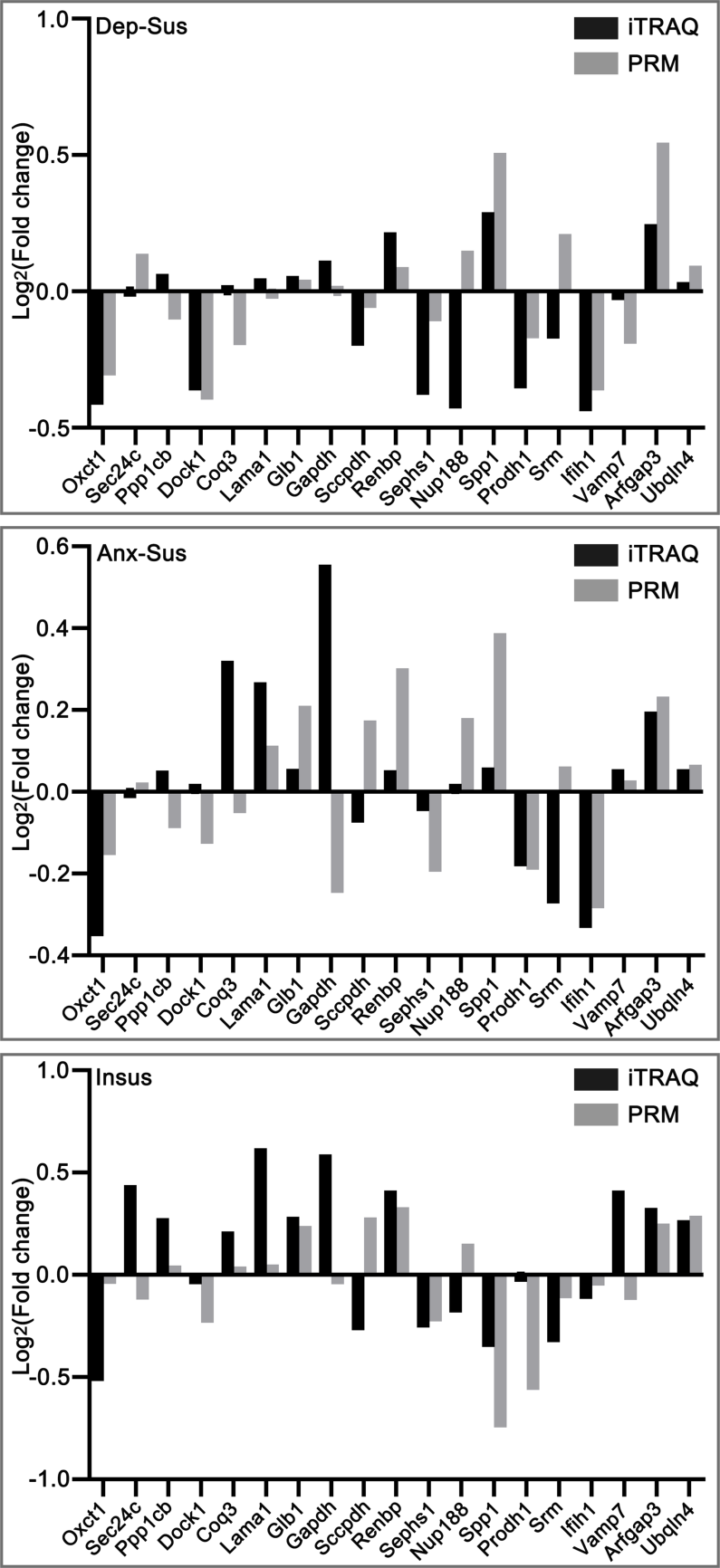


**Supplementary Figure S2**
